# Calorie Restriction Protects against Contrast-Induced Nephropathy via SIRT1/GPX4 Activation

**DOI:** 10.1155/2021/2999296

**Published:** 2021-10-19

**Authors:** Dandong Fang, Yongbin Wang, Ziyue Zhang, Donghai Yang, Daqian Gu, Bo He, Xiaoqun Zhang, Duofen He, HongYong Wang, Pedro A. Jose, Yu Han, Chunyu Zeng

**Affiliations:** ^1^Department of Cardiology, Daping Hospital, The Third Military Medical University, Chongqing, China; ^2^Chongqing Institute of Cardiology& Chongqing Key Laboratory of Hypertension Research, Chongqing, China; ^3^Cardiovascular Research Center of Chongqing College, Department of Cardiology of Chongqing General Hospital, University of Chinese Academy of Sciences, Chongqing, China; ^4^Division of Renal Disease & Hypertension, The George Washington University School of Medicine & Health Sciences, Washington, DC, USA

## Abstract

Calorie restriction (CR) extends lifespan and increases resistance to multiple forms of stress, including renal ischemia-reperfusion (I/R) injury. However, whether CR has protective effects on contrast-induced nephropathy (CIN) remains to be determined. In this study, we evaluated the therapeutic effects of CR on CIN and investigated the potential mechanisms. CIN was induced by the intravenous injection of iodinated contrast medium (CM) iopromide (1.8 g/kg) into Sprague Dawley rats with normal food intake or 40% reduced food intake, 4 weeks prior to iopromide administration. We found that CR was protective of CIN, assessed by renal structure and function. CM increased apoptosis, reactive oxygen species (ROS), and inflammation in the renal outer medulla, which were decreased by CR. The silent information regulator 1 (SIRT1) participated in the protective effect of CR on CIN, by upregulating glutathione peroxidase 4 (GPX4), a regulator of ferroptosis, because this protective effect was reversed by EX527, a specific SIRT1 antagonist. Our study showed that CR protected CIN via SIRT1/GPX4 activation. CR may be used to mitigate CIN.

## 1. Introduction

Contrast-induced nephropathy (CIN) is the abrupt deterioration of renal function resulting from intravenous or intra-arterial administration of iodinated contrast media used in diagnostic coronary angiography or percutaneous coronary intervention [1, 2]. The frequency of CIN ranges from 2% in low-risk patients to 50% in high-risk patients. It is reported to be the third leading cause of hospital-acquired acute renal injury. CIN is associated with increased morbidity, extended length of hospital stays, and increased costs [3–5]. The precise mechanism underlying CIN is not fully understood. A decrease in renal blood flow, increased reactive oxygen species (ROS) formation, and direct toxic affection renal tubule cells may be involved in the pathogenesis of CIN [6, 7].

There is no specific treatment for established CIN; prevention is the best strategy [8]. Attempts of preventing acute kidney injury with various substances such as N-acetylcysteine, vitamin C, statins, and furosemide have shown variable results. Adequate hydration is considered the basis of all preventive strategies [9]. However, a recent randomized trial found that prophylactic hydration has no benefit in protecting against CIN in patients with preexisting chronic kidney disease; even hydration by itself sometimes leads to complications [10]. There are 6–12 million high-risk patients, including those with preexisting chronic kidney disease, diabetes mellitus, and old age, undergoing procedures with intravascular iodinated contrast administration every year, worldwide [10]. Thus, it is urgent to identify novel preventive measures, especially without side effects; to decrease CIN incidence; and to improve clinical prognosis.

Calorie restriction (CR), via restriction of food intake, is the principal nongenetic or environmental intervention that extends lifespan without significant side effects [11]. Although the mechanisms of CR are complex, the prevention of oxidative stress and maintenance of antioxidant reserves are common features [12, 13]. CR has been proved to affect processes involved in cellular senescence, energy metabolism, stress signaling pathways, and formation of ROS [14]. There is accumulating evidence to indicate that silent information regulator 1 (SIRT1) mediates the effects of CR on metabolic reprogramming at a cellular level [15]. In addition to extending lifespan, CR has also been shown to provide protection against diverse acute tissue injuries, such as myocardial ischemia-reperfusion (I/R) injury [16] and cerebral I/R injury [17]. Mitchell and coworkers showed that a 30% reduction in daily calorie intake over short time periods prevented acute kidney injury in a murine I/R injury model [18]. Similar results have been reported in other models of renal injury [19]. However, whether CR has beneficial effects on CIN remains unclear. The aim of the study was to investigate whether the pretreatment with CR could mitigate CIN in rats and whether the renoprotective effects of CR are mediated by SIRT1 and its downstream pathway.

## 2. Methods and Materials

### 2.1. Animals

Adult male Sprague Dawley (SD) rats, weighting 230 ± 15 g, were obtained from the Laboratory Animal Center of Daping Hospital. All procedures were performed in accordance with institutional regulations and with the approval of the Experimental Animals Committee of Daping Hospital. The calorie-restricted rats (CR group) were fed 60% of the amount of food consumed by the control group for 4 weeks. Control rats had free access to food and water.

### 2.2. CIN Model

All the rats were deprived of water 24 h before the induction of CIN or mock CIN. Then, the rats were anesthetized with an intraperitoneal injection of pentobarbital sodium (50 mg/kg) and placed on a heating pad to maintain a constant body temperature of 37°C during the surgery. The left external jugular vein was cannulated with PE-10 tubing. SRT1720 (500 mg/kg), SIRT1 inhibitor EX527 (500 mg/kg), or ferrostatin-1 (Fer-1) (5 mg/kg), dissolved in sterile saline solution, was administrated intravenously, followed by the intravenous injection of contrast medium (CM) (iopromide, 1.8 g/kg). Then, the incisions were sutured, and the rats were allowed to recover for 48 h with free access to food and water. The rats were kept in metabolic cages for 24 h urine collections. The rats were reanesthetized with sodium pentobarbital (50 mg/kg body) at indicated times after CM injection. Then, a laparotomy and nephrectomy were performed. Blood samples obtained from abdominal aorta and the harvested kidneys were stored at −80°C for subsequent analyses.

### 2.3. Renal Function

The collected blood samples were centrifuged, and the sera were immediately analyzed with commercial kits. The renal function of the rats was assessed by measuring serum creatinine (Cr), blood urea nitrogen (BUN), and creatinine clearance (CCr). Serum samples were analyzed using an automated Beckman Analyzer (Beckman Instruments GmbH, Munich, Germany). Urine creatinine was measured using a Hitachi multianalyzer (Hitachi, 205D; Hitachinaka, Japan). CCr was calculated according to the formula CCr = *UV*/*P*, where *U* is the urinary Cr concentration (*μ*mol/L); *V* is the total urine volume collected for 24 h (mL/min); and *P* is the serum Cr concentration (*μ*mol/L) [20].

### 2.4. Renal Histopathology

The kidney samples were fixed and then dehydrated in increasing concentrations of ethanol. Subsequently, they were cleared in xylene and embedded in paraffin. The samples were cut into 4 *μ*m thick sections, followed by a staining with haematoxylin and eosin. Morphological damages to the kidney were scored to evaluate the degree of renal damage. These histopathologic damages included tubular epithelial cell swelling and vacuolization, cast formation, and desquamation. Pathological scoring, based on the presence of acute necrosis in proximal tubule cells, was quantified by the calculation of the percent of tubules that displayed cell necrosis, loss of brush border, cast formation, and tubule dilatation. The scale (1-5 points) is as follows: 0, none; 1, 0-10%; 2, 11-25%; 3, 26-45%; 4, 46-75%; and 5, 76-100%, as described previously [21]. At least 7 fields were examined in each slide.

### 2.5. Apoptosis of CM-Injured Kidney

Apoptotic cell death of kidney was detected using TUNEL assay [22]. In brief, tissue sections, selected from the outer medulla of the kidney, were heated at 65°C, washed in xylene, and dehydrated through a graded series of ethanol solutions. Next, sections were incubated in 10 *μ*g/mL protease K (Sigma, St Louis, MO) for 30 min at 37°C and then with 0.5% Triton X-100 for 10 min. Slides were rinsed twice with PBS and then incubated in 50 *μ*L of TUNEL reaction mixture for 60 min at 37°C in a humidified atmosphere in the absence of light. After PBS rinses, the tissue sections and cells were mixed with immunostaining blocking solution for 1 hat room temperature to prevent nonspecific antibody binding. A fluorescein in situ cell death detection kit was used according to the manufacturer's instructions for TUNEL assay (Roche Applied Science, Mannheim, Germany). Positive cells were counted in ten nonoverlapping fields of the outer medulla in each sample (magnification, ×400). The activity of caspase 3 in the renal outer medulla was measured using a Caspase 3 Colorimetric Assay Kit, following the manufacturer's protocol (Beyotime, China).

### 2.6. Measurement of DHE, MDA, SOD, GSH/GSSG, MPO, IL-*β*, and TNF-*α*

Oxidative stress of the injured kidney was evaluated by measuring the dihydroethidium (DHE), malondialdehyde (MDA), and superoxide dismutase (SOD) levels and the reduced and oxidized glutathione ratio (GSH/GSSG). The rat kidney tissues were quickly frozen, cut to a thickness of 8 *μ*m at an optimized cutting temperature, and mounted on glass slides [23]. The concentration of DHE in the renal outer medulla was calculated by the fluorescence intensity using ImageJ, according to the manufacturer's protocol (Beyotime Institute of Biotechnology, China). The renal outer medulla was homogenized in ice-cold sucrose buffer (pH 7.4) for the determination of MDA, SOD, GSH, myeloperoxidase (MPO), IL-1*β*, and tumor necrosis factor-*α* (TNF-*α*) levels. The concentration of MDA in the renal outer medulla was calculated using the thiobarbituric acid method, according to the manufacturer's protocol (Assay Kit from Nanjing Jiancheng Bioengineering Institute, Nanjing, China). Total SOD activity was evaluated using the nitroblue tetrazolium method according to the manufacturer's instructions (Beyotime Institute of Biotechnology, China). SOD activity was expressed as units/mg protein. The intracellular levels of GSH were measured using GSH and GSSG Assay Kit (Beyotime Institute of Biotechnology, China). MPO, IL-1*β*, and TNF-*α* were used as a marker of inflammatory response, and their levels were determined using ELISA kits (R&D Systems, Minneapolis, MN).

### 2.7. Western Blot Analysis

Kidney samples (40 mg) were ground and homogenized in ice-cold lysis buffer (20 mmol/L Tris-HCl, pH 7.4; 2 mmol/L EDTA, pH 8.0; 2 mmol/L EGTA; 100 mmol/L NaCl; 10 *μ*g/mL leupeptin; 10 *μ*g/mL aprotinin; 2 mmol/L phenylmethylsulfonyl fluoride; and 1% NP-40). The tissue homogenate was sonicated and kept on ice for one hour. The lysate was centrifuged at 15,000 × g for 40 min. The supernatant was collected, and the concentration of the protein was measured by using a protein assay kit (Bio-Rad Laboratories, Hercules, CA) whose standard was bovine serum albumin. The supernatants were boiled in a sample buffer (35 mmol/L Tris-HCl, pH 6.8; 4% SDS; 9.3% dithiothreitol; 0.01% bromophenol blue; and 30% glycerol) for 5 min at 95°C. The samples were stored at -20°C prior to use. Protein samples (50 mg) were separated by SDS-PAGE with 8% polyacrylamide gel and electrotransferred to nitrocellulose membranes. After the nonspecific binding sites were blocked in TBS (Tris-buffered saline) containing 5% nonfat dry milk for 1 hour, the membranes were incubated with primary antibodies with recommended dilution of anti-SIRT1 (Millipore, Bedford, MA; dilution 1 : 500), anti-GPX4 (Proteintech; dilution 1 : 500), anti-total caspase-3, and anti-cleaved caspase-3 (Cell Signaling Technology; dilution 1 : 1000) at 4°C overnight. GAPDH (Santa Cruz Biotechnology; dilution 1 : 1000) were used as housekeeping proteins. The membranes were then incubated with the diluted (1: 15000) secondary antibody (Li-Cor Biosciences, Lincoln, NE) at room temperature for 1 hour. Membranes were washed three times with TBST, and the bounds were detected by the Odyssey Infrared Imaging System (Li-Cor Biosciences, Lincoln, NE). The images were analyzed using the Odyssey Application Software to obtain the integrated intensities (Figure S3 in the Supplementary Material). ImageJ was used to calculate the integrated intensity of the western blots.

### 2.8. Analysis of Plasma Iodine

Plasma iodine was measured using the Sandell-Kolthoff method [24]. Blood was drawn from the abdominal aorta of rats 15 min after CM injection. The plasma was separated from clotted blood by centrifugation. 0.5 mL perchloric acid and 0.6 mL sodium chlorate solutions were added to 0.1 mL plasma, mixed, and placed in a digestion-controlled heating device at 130°C for 120 min. Then, the mixture was removed and cooled at 20°C. 3 mL arsenious acid was added to the just-cooled sample (about 1.2 mL), fully mixed, and allowed to react for 15 min. Add 0.6 mL of ammonium cerium sulfate solution to each tube (about 4.8 mL) in sequence at the same time interval (30 s or 20 s). When the absorbance value of the standard tube 300 *μ*g/L iodine concentration tube reached about 0.10, the absorbance value of each tube was measured. When the iodine concentration in plasma exceeded the measurement threshold, the plasma was diluted accordingly, before the iodine concentration was measured.

 $$\begin{array}{c} {\text{H}}_{3}{\text{AsO}}_{3}+2\text{C}{\text{e}}^{4+}+{\text{H}}_{2}\text{O}\overset{{\text{I}}^{-}}{⟶}{\text{H}}_{3}{\text{AsO}}_{4}+2\text{C}{\text{e}}^{3+}+2{\text{H}}^{+} \end{array}$$

### 2.9. Statistical Analysis

Data are presented as mean ± standard deviation. One-way factorial ANOVA, followed by Dunnett's post hoc test, was used for multigroup (>2) comparison. *P* < 0.05 was considered significant.

## 3. Results

### 3.1. CR Protects Renal Injury in CIN

**Figure 1:**
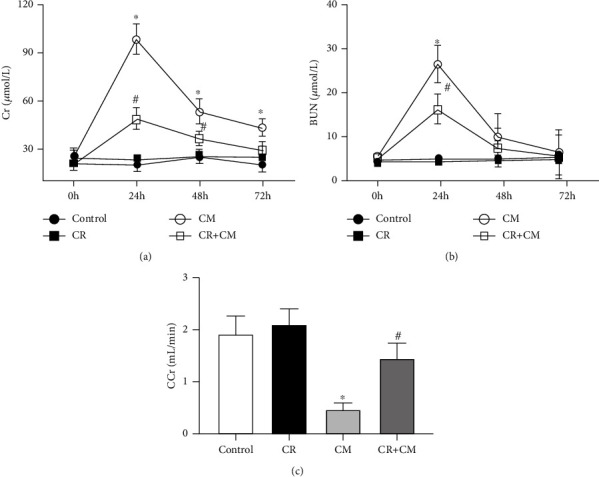
Effect of CR on renal function in SD rats with CIN. SD rats were pretreated with CR for 4 weeks. CIN was induced by the intravenous injection of the CM iopromide (1.8 g/kg). Serum samples were collected at the indicated times after CM injection. Serum Cr (a) and BUN (b) were measured 24 h, 48 h, and 72 h after CM injection. (c) CCr was measured 24 h after CM injection. The values are presented as mean ± standard deviation (*n* = 15, ^∗^*P* < 0.05 vs. control and ^#^*P* < 0.05 vs. CM alone).

The rats were studied after 4 weeks of after control diet or CR. The rats after CR (263.8 ± 6.1 g) lost about 15 percent of body weight, relative to the control group (309.1 ± 7.9 g) (Figure S1(a) in the Supplementary Material). There were no significant differences in plasma cholesterol and glucose levels between two groups (Figures S1(b) and S1(c) in the Supplementary Material). Because the CR mice lost 15% of their body weight and considering the relationship between blood volume and body weight [25–28], we measured the plasma iodine concentration 15 min after CM injection. There were no significant differences in plasma iodine concentration between the two groups (Figure S1(d) in the Supplementary Material) (control group, 1.72 ± 0.23 mg/mL vs. the CR group, 1.62 ± 0.31 mg/mL). The intravenous injection of CM (iopromide, 1.8 g/kg) caused CIN, proved by the increase in BUN and serum Cr levels and decrease in CCr of the CM group (Figures 1(a)-1(c)). By contrast, in the CR group, relative to the control group, the CM-induced increases in BUN and serum Cr and decrease in CCr were less (Figures 1(a)-1(c)), indicating the protection of CIN by CR.

**Figure 2:**
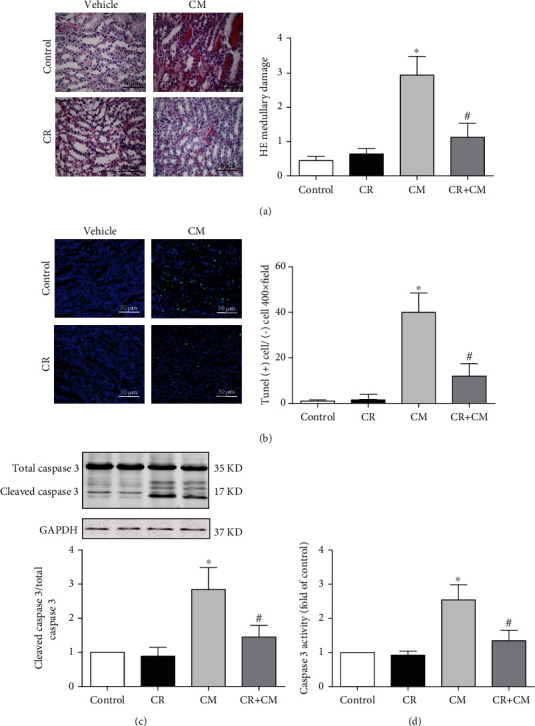
Effect of CR on apoptosis of renal outer medulla in SD rats with CIN. SD rats were pretreated with CR for 4 weeks. CIN was induced by the intravenous injection of the CM iopromide (1.8 g/kg). The kidney samples were collected 24 h after CM injection. (a1) HE staining of renal outer medulla (scale bar = 50 *μ*m). (a2) Pathological scores. (b1) TUNEL staining of renal outer medulla (scale bar = 50 *μ*m) and (b2) TUNEL-positive cells counted in 10 high-power (400×) fields. (c) Western blot analysis of cleaved caspase 3 (a marker of apoptosis), total caspase-3 expression, and cleaved caspase 3/total caspase 3 ratio. The values are presented as fold-change of control group. (d) Caspase-3 activity detected by ELISA. The values are fold change of the control group; each control sample is given a value of 1. The values are presented as mean ± standard deviation (*n* = 7, ^∗^*P* < 0.05 vs. control, ^#^*P* < 0.05 vs. CM alone).

Consistent with our previous report [29], CIN was characterized by marked proximal tubule dropout, extensive interstitial inflammation, and extensive intratubular cast formation 24 h after CM injection. The main lesion was in the outer medulla. The abovementioned pathological changes were reduced by CR, proved by the reduction in renal pathological scores in HE-stained kidneys (Figure 2(a)).

Apoptosis is one of the major CM-induced cytotoxic effects on the kidney [30]. We found that CM increased apoptosis, determined by TUNEL staining, in the boundary between the cortex and medulla. The TUNEL-positive cells were decreased approximately three times in the CR+CM group, relative to the CM group (Figure 2(b)). Consistent with the decrease in TUNEL-positive cells in the CR+CM group, relative to the CM group, the cleaved caspase 3 protein expression and caspase 3 activity were also elevated in CIN, which were also attenuated by CR pretreatment (Figures 2(c) and 2(d)). Taken together, CR protects from CIN by attenuation of apoptosis.

### 3.2. The Protective Effect of CR on CIN Is Mediated by SIRT1 Activation

**Figure 3:**
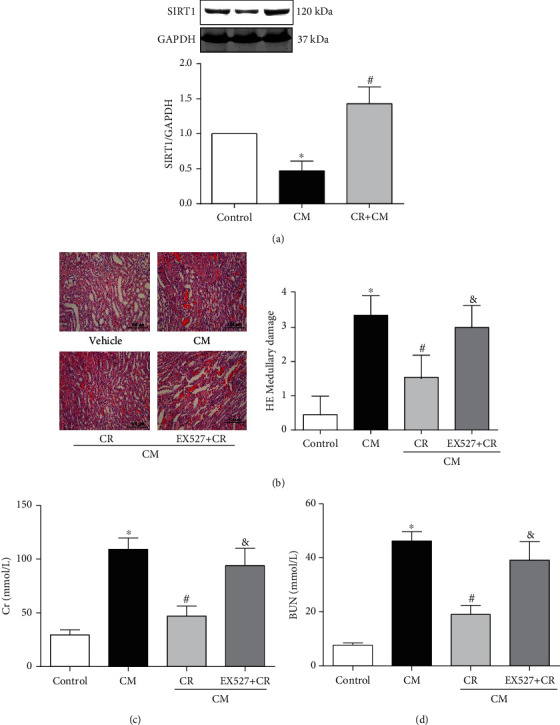
Role of SIRT1 in the renal protective effect of CR on CIN. SD rats were pretreated with CR for 4 weeks. CIN was induced by the intravenous injection of the CM iopromide (1.8 g/kg). The SIRT1 inhibitor EX527 (500 mg/kg) was injected intravenously before establishment of CIN. The kidney samples were collected 24 h after CM injection. (a) Western blot analysis of SIRT1 expression in kidney tissues. Values are presented with fold change of the control group; each control sample is given a value of 1. (b1) HE staining of renal outer medulla (scale bar = 100 *μ*m). (b2) Pathological scores are shown. Serum Cr (c) and BUN (d) levels were measured in different groups, 24 h after CM injection. The values are presented as mean ± standard deviation (*n* = 7, ^∗^*P* < 0.05 vs. control, ^#^*P* < 0.05 vs. CM alone, ^&^*P* < 0.05 vs. CM+CR).

**Figure 4:**
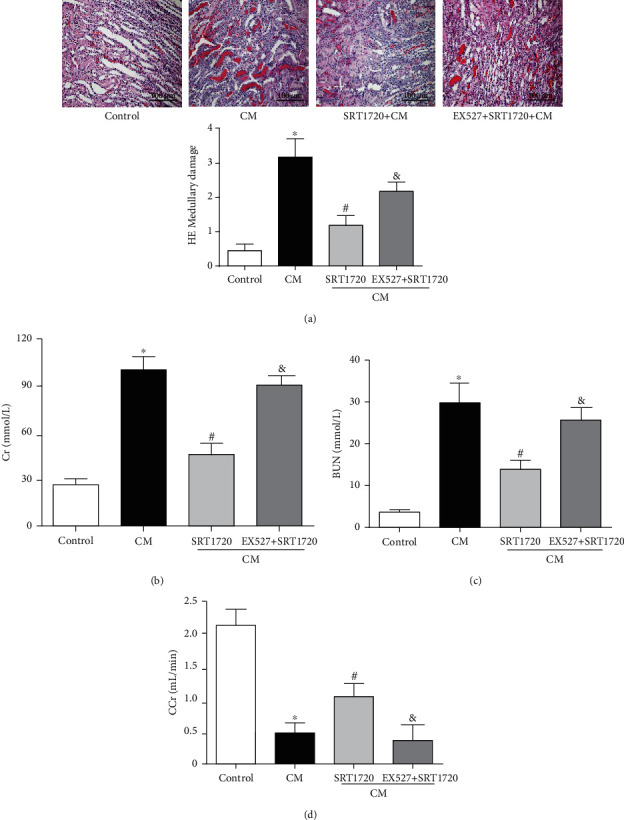
Activation of SIRT1 alleviates CIN. CIN was induced by the intravenous injection of the CM iopromide (1.8 g/kg). The SIRT1 inhibitor EX527 (500 mg/kg) or/and SIRT1-specific activator SRT1720 (500 mg/kg) were injected intravenously before establishment of CIN. The kidney samples were collected 24 h after CM injection. (a1) Representative HE staining of kidney sections and (a2) pathological scores. Serum Cr (b) and BUN (c) levels of rats were measured 24 h after CM injection. (d) CCr was measured 24 h after CM injection. The values are presented as mean ± standard deviation (*n* = 7, ^∗^*P* < 0.05 vs. control, ^#^*P* < 0.05 vs. CM alone, and ^&^*P* < 0.05 vs. CM+SRT1720).

Previous studies indicated the role of SIRT1 in the protection of CR in various diseases, including diabetes [31, 32] and neurodegenerative diseases [33]. Therefore, we wondered whether or not SIRT1 was involved in the protective effect of CR on CIN. Our study showed that SIRT1 expression, determined by immunoblotting, was lower in the CM group, compared with that control group, which was reversed by CR (Figure 3(a)). In the presence of EX527 (500 mg/kg), a specific SIRT1 antagonist, CR-mediated protection on CIN was lost, proved by the higher renal HE scores, serum Cr, and BUN in the CM+EX527+CR than the CM+CR group (Figures 3(b)–3(d)). Moreover, the protective effect of CR on CIN was simulated by SRT1720 (500 mg/kg), a SIRT1-specific activator. Pretreatment with the SIRT1 activator SRT1720 mitigated the renal proximal tubule dropout in CIN, detected by renal HE scores, an effect that was blocked by the SIRT1 antagonist EX527 (Figure 4(a)). Moreover, the protective effect of SRT1720 in CIN was also demonstrated by improvement of renal function, shown by the lower serum Cr and BUN levels and higher CCr (Figures 4(b)–4(d)); these effects were blocked by the SIRT1 antagonist EX527.

### 3.3. Role of SIRT1-Mediated Antioxidants, Antiferroptosis, and Anti-Inflammation in the Renal-Protective Effect of CR on CIN

**Figure 5:**
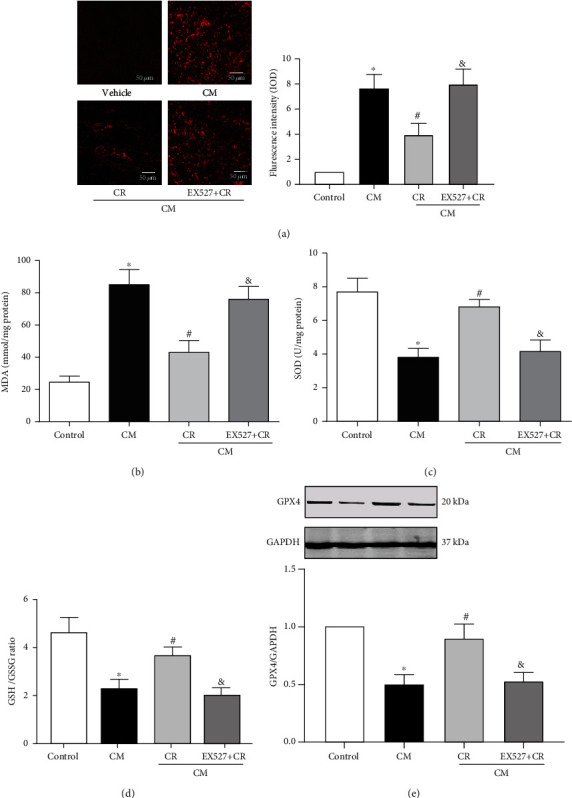
Role of SIRT1 in the antioxidants and antiferroptosis effects of CR on CIN. SD rats were pretreated with CR for 4 weeks. CIN was induced by the intravenous injection of the CM iopromide (1.8 g/kg). The SIRT1 inhibitor EX527 (500 mg/kg) was injected intravenously before establishment of CIN. The kidney samples were collected at 24 h after CM injection. (a1) Tissue oxidative stress level in the renal outer medulla, measured using DHE (scale bar = 50 *μ*m). (a2) The quantitative mean fluorescence intensity in each treatment group is shown. The values are presented with fold change of the control group. The renal levels of MDA (b), SOD (c), and GSH/GSSG ratio (d) were measured to determine the oxidative stress. (e) Western blots and relative band densities of GPX4 (regulator of ferroptosis) in the renal outer medulla of different groups. The values are presented as fold change of the control group. The values are presented as mean ± standard deviation (*n* = 7, ^∗^*P* < 0.05 vs. control, ^#^*P* < 0.05 vs. CM alone, and ^&^*P* < 0.05 vs. CM+CR).

As an antioxidant, SIRT1 plays a protective role in many diseases, via downregulation of ROS formation. Our present study showed that CM increased ROS levels, determined by measurement of DHE, MDA, SOD, and GSH levels [34]. Pretreatment with CR decreased the CM-induced increase in DHE and MDA production (Figures 5(a) and 5(b)) while it increased the CM-induced decrease on SOD and GSH levels in the renal outer medulla (Figures 5(c) and 5(d)). In addition, the protective effect of CR on ROS formation was lost in the presence of the SIRT1 antagonist EX527.

Accumulation of ROS and cell death are reminiscent of ferroptosis [35]. Whereas glutathione peroxidase 4 (GPX4), a regulator of ferroptosis, was decreased after injection of CM, CR alleviated the reduction of GPX4 in CIN that was blocked by EX527 (Figure 5(e)).

Since there is a close relationship between ROS and inflammation, we also checked the inflammation induced by CM by determination of the renal outer medulla MPO, IL-1*β*, and TNF-*α* protein levels. Consistent with the ROS levels, CM increased MPO, IL-1*β*, and TNF-*α* levels that were decreased by CR. In the presence of EX527, the anti-inflammatory effect of CR in CIN was lost (Figures S2(a)–S2(c) in the Supplementary Material).

### 3.4. Inhibition of Ferroptosis Prevents Renal Injury in CIN

**Figure 6:**
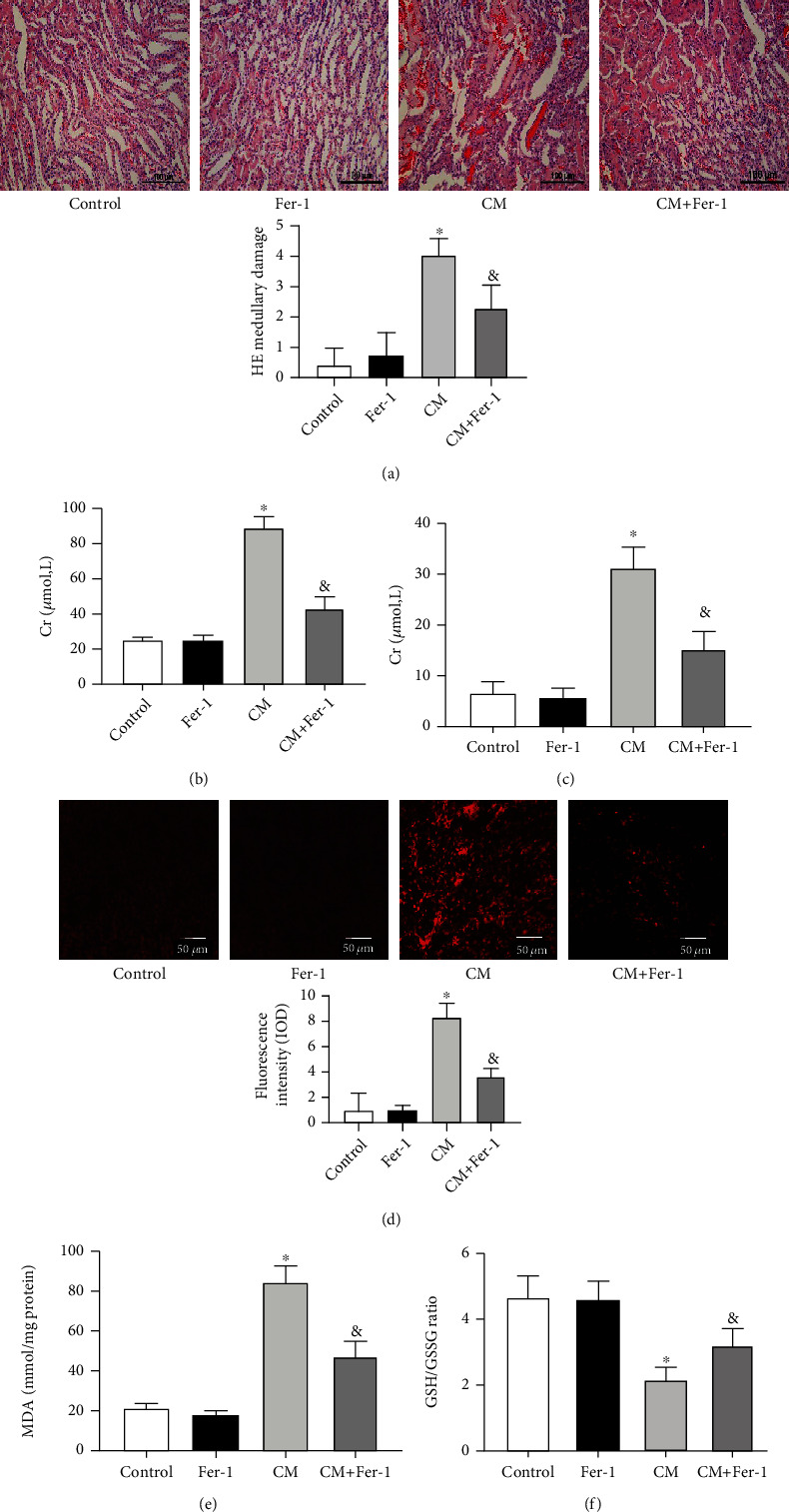
Fer-1 prevented renal injury in CIN. SD rats were pretreated with Fer-1 for 30 min, followed by injection of CM and euthanasia at 24 h. (a1) Fer-1 decreased histologic injury in kidneys from rats exposed to CM (scale bar = 100 *μ*m). (a2) Pathological scores were calculated. Serum Cr (b) and BUN (c) levels were measured 24 h after CM injection. (d1) Tissue oxidative stress level in the renal outer medulla, measured using DHE (scale bar = 50 *μ*m). (d2) The quantitative mean fluorescence intensity in each treatment group is shown. The renal levels of MDA (e) and GSH/GSSG ratio (f) were measured to determine the oxidative stress. The values are presented as mean ± standard deviation (*n* = 7, ^∗^*P* < 0.05 vs. control, ^&^*P* < 0.05 vs. CM).

Many studies have found that ferroptosis plays an important role in kidney diseases [36, 37]. Pretreatment of Fer-1 (5 mg/kg), an inhibitor of ferroptosis, improved renal function in CIN, assessed by HE scores (Figure 6(a)), serum Cr (Figure 6(b)), and BUN (Figure 6(c)). Moreover, we found that Fer-1 could reduce DHE intensity and MDA level (Figures 6(d) and 6(e)) and increase GSH levels (Figure 6(f)) in CIN. Therefore, we confirmed that Fer-1 could reduce the total ROS production in CIN. Therefore, ferroptosis plays an important role in CIN, and inhibition of ferroptosis can inhibit the renal damage caused by CM.

## 4. Discussion

Although the exact mechanisms that cause CIN are not clear, oxidative stress and inflammation are two major factors that play pivotal roles in the development of CIN. The generation of excessive levels of ROS under hypoxia can directly cause endothelial dysfunction, renal tubular damage, and abnormal tubular transport [38, 39]. Reactive oxygen species imbalance also causes lipid peroxidation that leads to cytotoxic damage [40]. In addition, activation of cytokine-induced inflammatory mediators by reactive free radicals exacerbates the cellular injury [41]. In the present study, increased MDA and decreased SOD and GSH levels were observed in CIN; increased MPO, IL-1*β*, and TNF-*α* levels were also found.

CR, a proven powerful strategy to expand lifespan in many animals, was reported to exert protection in chemotherapeutic drug-induced injury [42]. Nutrient signaling activated by CR plays important roles in protection from injury. For example, CR combined with resveratrol, which has antioxidant properties, protects the rat heart from doxorubicin-induced toxicity [43]. CR also enhances cellular adaptation to hypoxia in aged kidneys [44] and ameliorates age-induced structural and functional changes, such as glomerulosclerosis, tubulointerstitial fibrosis, decline in renal blood flow, and loss of several tubular transport functions [45]. CR, a nongenetic and environmental intervention that is free from side effects, may benefit high-risk patients who undergo procedures involving intravascular iodinated contrast administration. In our study, rats with CIN caused by CM and fed a CR diet had a better renal function and structure than the CM group on normal caloric intake, suggesting a protective role of CR in CIN. Indeed, the degree of apoptosis, oxidative stress, and inflammation was attenuated in the CR-treated CM group, relative to the CM group on normal caloric intake, which further confirmed the protective effect of CR in CIN.

Many studies showed that CR is associated with the alterations in energy metabolism. SIRT1, a NAD^+^-dependent deacetylase, is one of the seven mammalian sirtuins and is known to play an important role in maintaining metabolic homeostasis in many tissues [12, 46]. There are several reports on SIRT1-mediated protection from injury in the brain, kidney and other organs [17, 47, 48]. Relevant to our present study, a previous study elucidated on the ability of SIRT1 activation to protect the renal medulla from oxidative injury in mice [49]. Our present study demonstrated that SIRT1 expression in the renal outer medulla was significantly reduced in CIN. In mice pretreated with CR, renal SIRT1 expression was increased and CIN was attenuated, which showed a protective effect of SIRT1 on CIN *in vivo*. Furthermore, the protective effect of CR on CIN was mimicked by SRT1720, a SIRT1-specific activator. In the presence of EX527, a specific SIRT1 antagonist, the CR-mediated protection on CIN was lost, confirming that CR-mediated protection from contrast-induced nephropathy occurs via SIRT1 activation.

The term ferroptosis was coined in 2012 [50] to describe the form of cell death induced by the small molecule elastin, which leads to GSH depletion and inactivation of the phospholipid peroxidase GPX4 [51]. Recently, several studies have found that ferroptosis plays a key role in kidney injury [36, 37, 52]. However, the relationship between ferroptosis and CIN is unclear. Our present study demonstrated that renal ferroptosis is an important pathological damage factor in CIN. Moreover, a recent study found that stabilization of the expression of SIRT1 can inhibit ferroptosis-induced cell death [53]. Our current study also found that CR could alleviate the reduction of renal GPX4 after injection of CM, an effect that was blocked by SIRT1 antagonist EX527. In addition, we showed that Fer-1, an inhibitor of ferroptosis, improved the renal function and reduced the oxidative stress in CIN, confirming the involvement of GPX4 in the beneficial effect of CR. However, there are still some questions that need to be answered. For example, how does SIRT1 regulate GPX4 in the kidney? The answer can probably give us a clue to explain the different functions of SIRT1 in different situations.

### 4.1. Limitation

We realize that the body weight difference between the CR and non-CR group in the calculation of the dosage of CM may influence the results of experiments. Our CR study was performed in SD rats, results of which may be influenced by other rat models, such as the Zucker rat [26], because the obese Zucker rat is much heavier than the control lean Zucker rat. By contrast, in our present study, the control SD rat weights of 309.1 ± 7.9 g were only 15% higher than CR SD rats with body weights of 263.8 ± 6.1 g. To determine if the absolute amount of injected CM affected the plasma CM concentration, we measured the plasma iodine concentration by the Sandell-Kolthoff method [24]. We found that there was no significant difference in plasma iodine concentration between the two groups (control group, 1.72 ± 0.23 mg/mL vs. the CR group, 1.62 ± 0.31 mg/mL). Moreover, a previous report [54] showed that iodine concentrations, after the unit conversion, from 3.5 to 7.0 g/kg did not affect renal function. To avoid the bias of CM dosage due to the body weight, it is better to ascertain first the plasma CM concentration by measurement of plasma iodine concentration, before doing subsequent experiments.

In conclusion, the present study demonstrated that CR, via SIRT1/GPX4, protects against CIN. CR may be a potential method to prevent CIN.

## 5. Conclusions

This study revealed a crucial role of CR in extent of CIN in SD rats. We are the first to show that CR can attenuate CIN through its antioxidant and anti-inflammatory effects, via the SIRT1/GPX4 pathway, in renal tissue. SIRT1/GPX4 could be a potential therapeutic target to prevent kidney injury after CM injection in patients.

## Data Availability

The research article data used to support the findings of this study are included within the article.

## Supplementary Materials

Supplementary Materials Figure S1: body weight and serum total cholesterol, blood glucose, and plasma iodine levels. (a) Body weights (g) of rats were checked during 4 weeks of CR. Serum samples of rats were collected to measure the levels of serum total cholesterol (b) and blood glucose (c) after 4 weeks of CR. (d) Plasma iodine (mg/mL) concentrations of the control, non-CR group, and CR group were measured 15 minutes after injection of CM. The values are presented as mean ± standard deviation (*n* = 7, ^∗^*P* < 0.05 vs. control). Figure S2: role of SIRT1 in the anti-inflammatory effects of CR in CIN. SD rats were pretreated with CR for 4 weeks. CIN was induced by intravenous injection of the CM iopromide (1.8 g/kg). The SIRT1 inhibitor EX527 (500 m/kg) was injected intravenously before establishment of CIN. The kidney samples were collected 24 h after CM injection. MPO activity (a) and IL-1*β* (b) and TNF-*α* (c) levels in the renal outer medulla were measured to determine the inflammatory response (^∗^*P* < 0.05 vs. control). The values are presented as mean ± standard deviation (*n* = 7, ^∗^*P* < 0.05 vs. control, ^#^*P* < 0.05 vs. CM alone, ^&^*P* < 0.05 vs. CM+CR). Figure S3: original western blot images. (a) and (b): refer to Figure 2(c). (c) and (d): refer to Figure 3(a). (e) and (f): refer to Figure 5(e).
