# Corrigendum to “Calorie Restriction Protects against Contrast-Induced Nephropathy via SIRT1/GPX4 Activation”

**DOI:** 10.1155/2022/9846101

**Published:** 2022-03-10

**Authors:** Dandong Fang, Yongbin Wang, Ziyue Zhang, Donghai Yang, Daqian Gu, Bo He, Xiaoqun Zhang, Duofen He, HongYong Wang, Pedro A. Jose, Yu Han, Chunyu Zeng

**Affiliations:** ^1^Department of Cardiology, Daping Hospital, The Third Military Medical University, Chongqing, China; ^2^Chongqing Institute of Cardiology& Chongqing Key Laboratory of Hypertension Research, Chongqing, China; ^3^Division of Renal Disease & Hypertension, The George Washington University School of Medicine & Health Sciences, Washington, DC, USA; ^4^Cardiovascular Research Center of Chongqing College, Department of Cardiology of Chongqing General Hospital, University of Chinese Academy of Sciences, Chongqing, China

In the article titled “Calorie Restriction Protects against Contrast-Induced Nephropathy via SIRT1/GPX4 Activation” [1], incorrect affiliation was given for the authors Pedro A. Jose and Chunyu Zeng. The corrected author list and affiliation is shown above.

Additionally, the Authors' Contributions section should read as follows:
