# Correction to “Calorie Restriction Protects against Contrast‐Induced Nephropathy via SIRT1/GPX4 Activation”

**DOI:** 10.1155/omcl/9848470

**Published:** 2025-12-16

D. Fang, Y. Wang, Z. Zhang, D. Yang, D. Gu, B. He, X. Zhang, D. He, H. Wang, P. A. Jose, Y. Han, and C. Zeng, “Calorie Restriction Protects against Contrast‐Induced Nephropathy via SIRT1/GPX4 Activation” *Oxidative Medicine and Cellular Longevity*, no. 2021 (2021). https://doi.org/10.1155/2021/2999296.

In the article titled “Calorie Restriction Protects against Contrast‐Induced Nephropathy via SIRT1/GPX4 Activation,” there was an error in Figure 4a related to an image of the incorrect tissue sample being placed in the CM group. The error was introduced by the authors during figure assembly and Figure 4 should be corrected as follows:

Figure 4Activation of SIRT1 alleviates CIN. CIN was induced by the intravenous injection of the CM iopromide (1.8 g/kg). The SIRT1 inhibitor EX527 (500 mg/kg) or/and SIRT1‐specific activator SRT1720 (500 mg/kg) were injected intravenously before establishment of CIN. The kidney samples were collected 24 h after CM injection. (a1) Representative HE staining of kidney sections and (a2) pathological scores. Serum Cr (b) and BUN (c) levels of rats were measured 24 h after CM injection. (d) CCr was measured 24 h after CM injection. The values are presented as mean ± standard deviation (*n* = 7, ^∗^ *P*  < 0.05 vs. control, ^#^ *P*  < 0.05 vs. CM alone, and ^&^ *P*  < 0.05 vs. CM + SRT1720).

**Figure:**
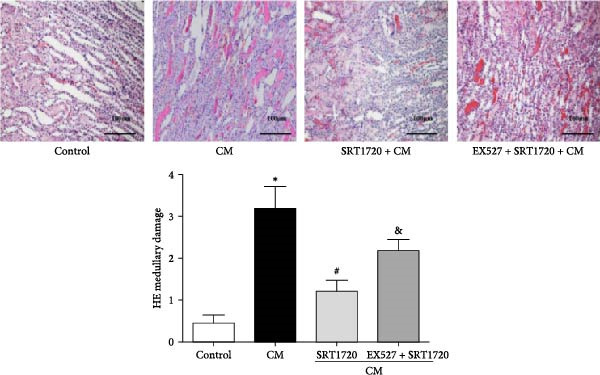
(a)

**Figure:**
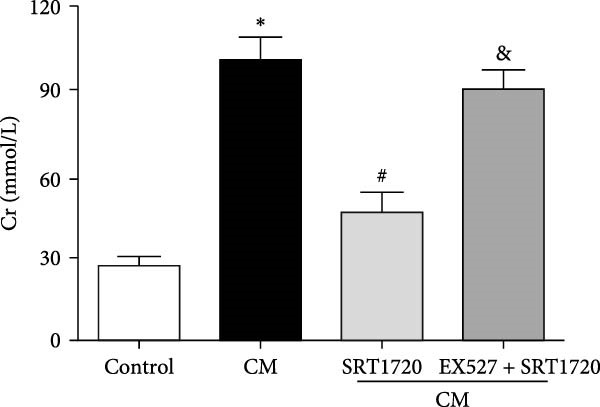
(b)

**Figure:**
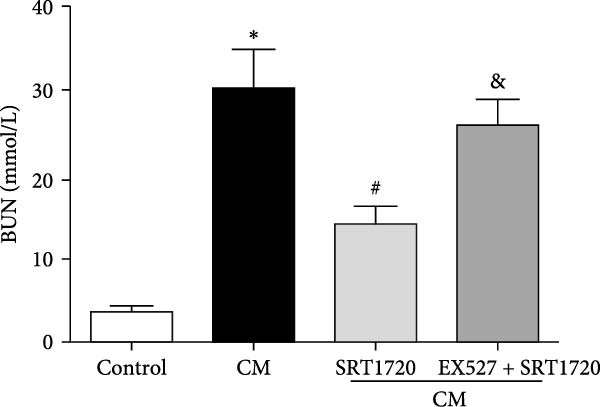
(c)

**Figure:**
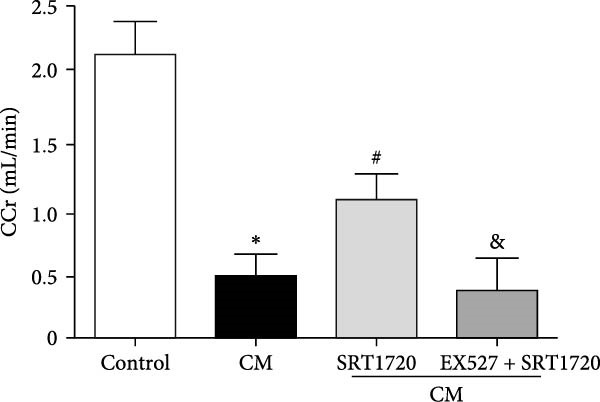
(d)

We apologize for this error.

