# Supplementary figures and images for: Calorie Restriction Protects against Contrast-Induced Nephropathy via SIRT1/GPX4 Activation
Source: Oxid Med Cell Longev. 2021 Oct 19;2021:2999296. doi: 10.1155/2021/2999296 (PMC8548166; doi:10.1155/2021/2999296)

Figure S1

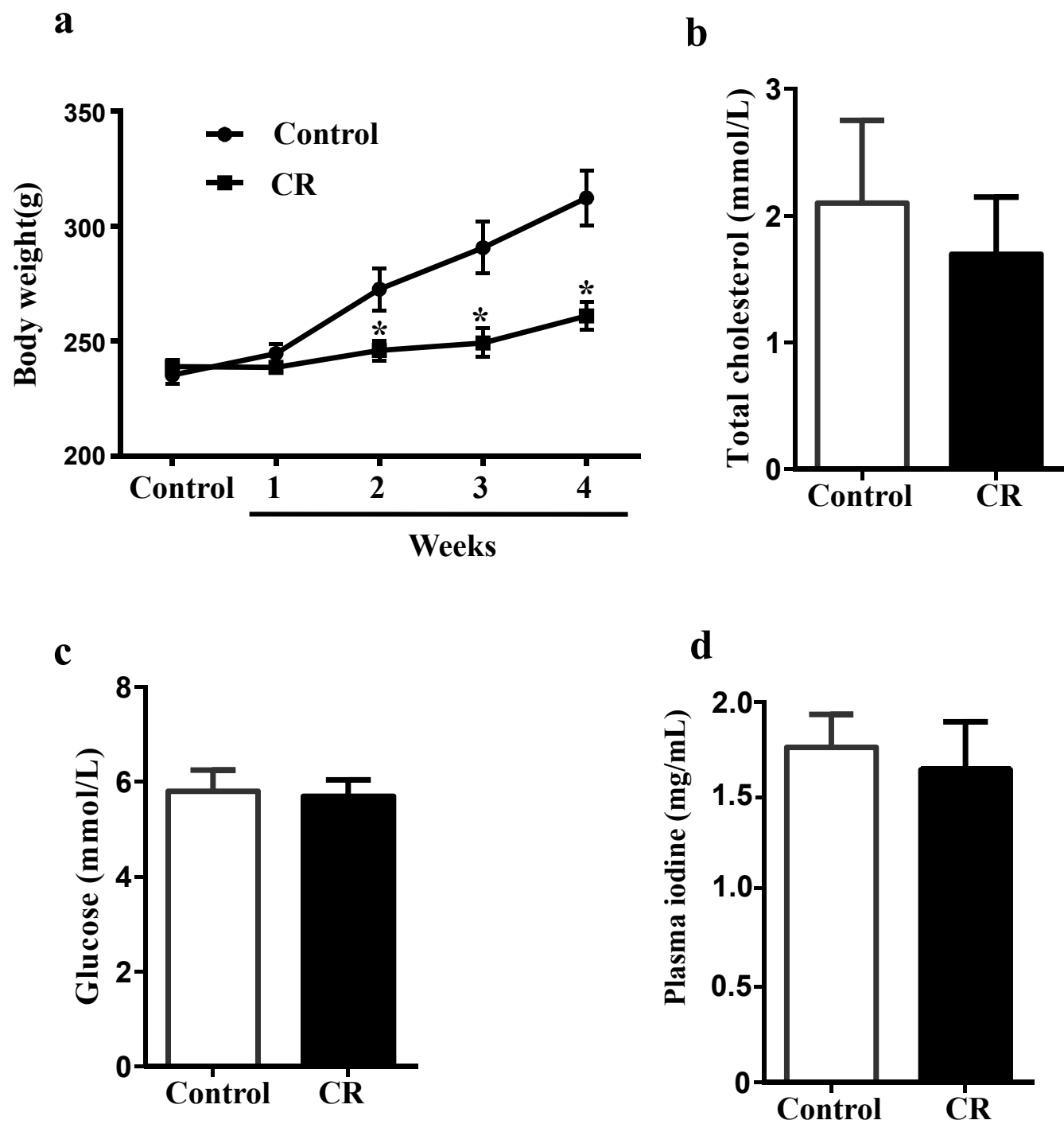

Figure S2

**a**

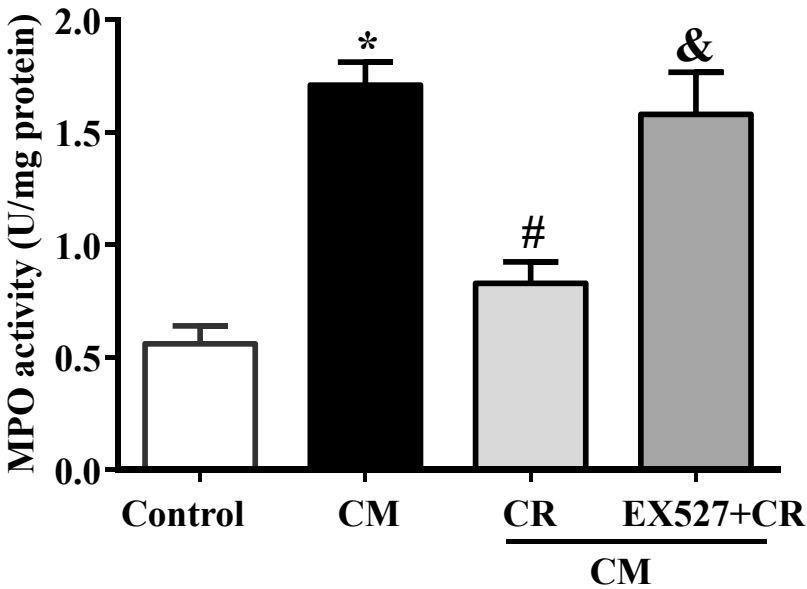

**b**

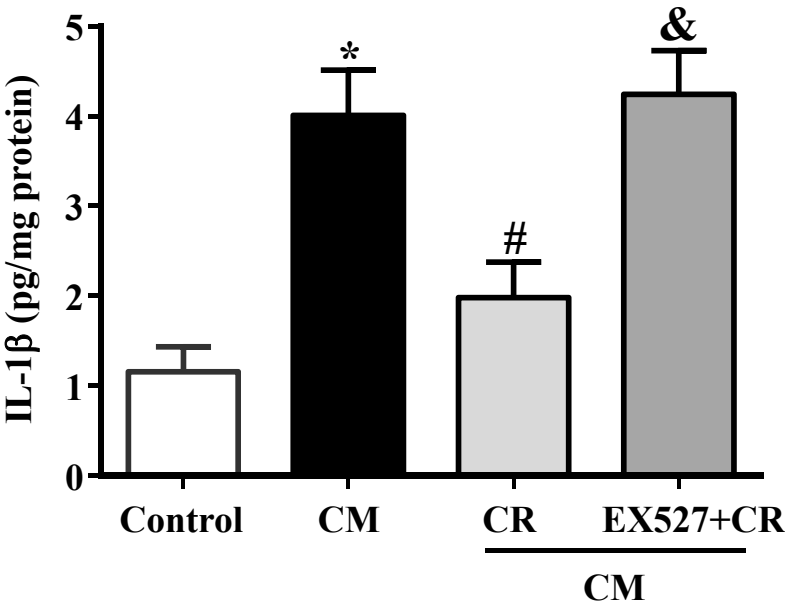

**c**

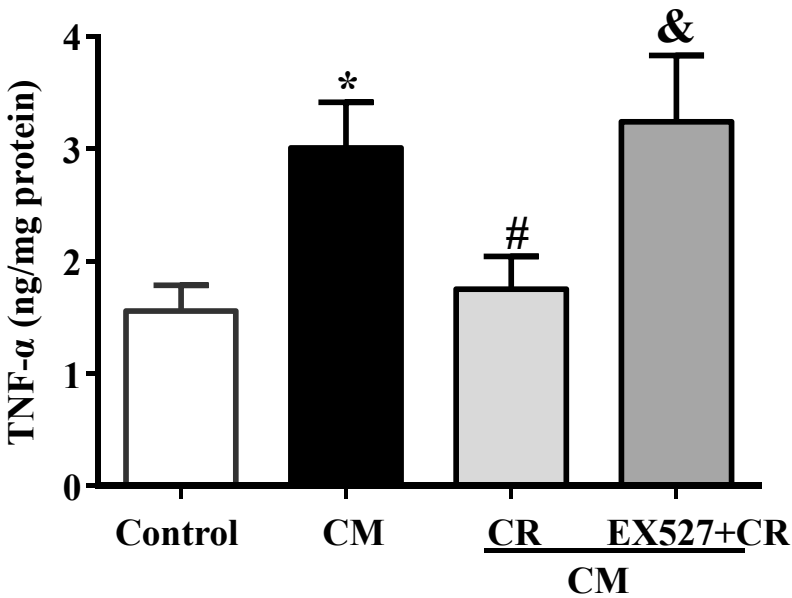

**Figure S3**

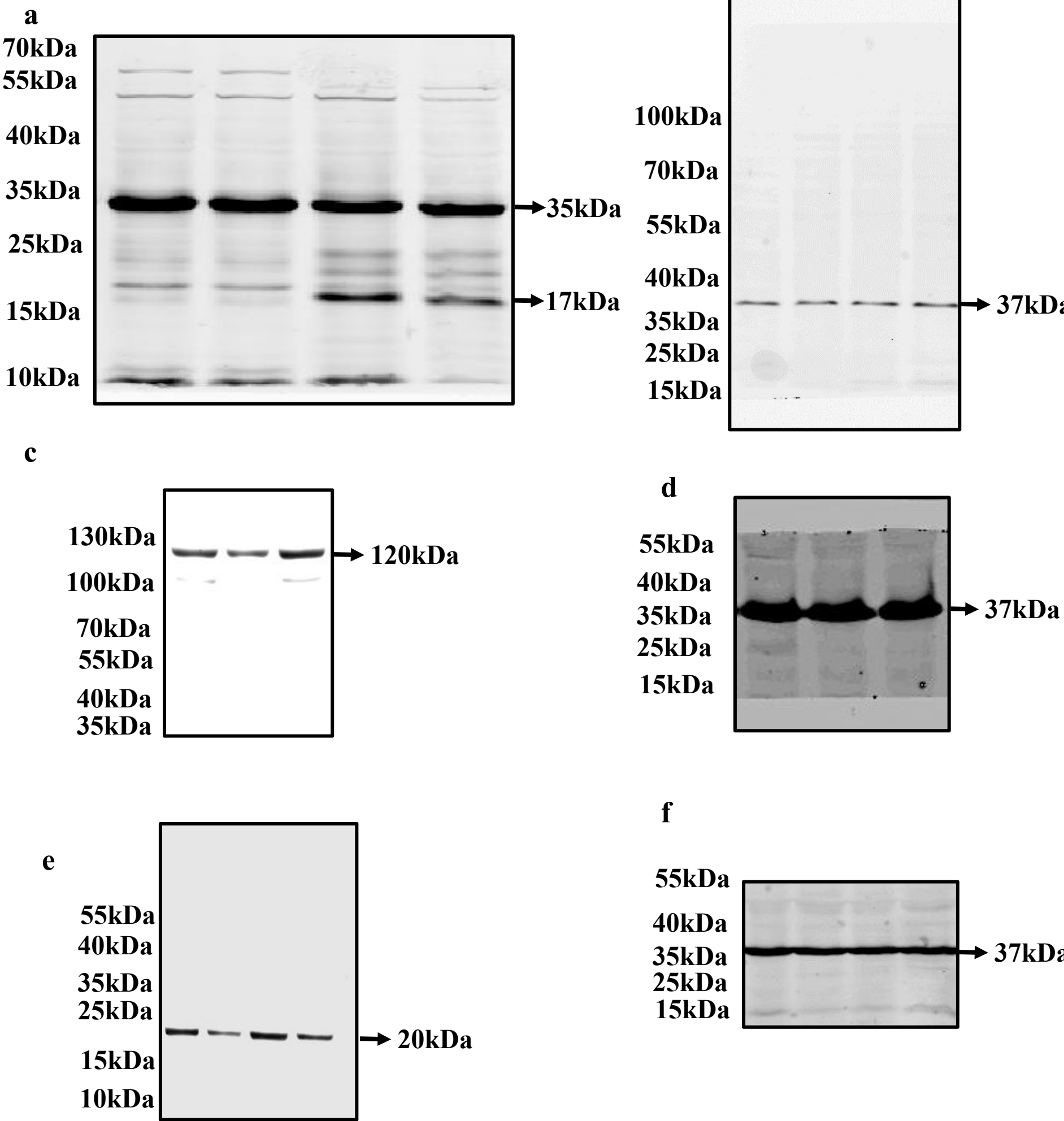

Supplement: Supplementary Materials — Figure S1: body weight and serum total cholesterol, blood glucose, and plasma iodine levels. (a) Body weights (g) of rats were checked during 4 weeks of CR. Serum samples of rats were collected to measure the levels of serum total cholesterol (b) and blood glucose (c) after 4 weeks of CR. (d) Plasma iodine (mg/mL) concentrations of the control, non-CR group, and CR group were measured 15 minutes after injection of CM. The values are presented as mean ± standard deviation (n = 7, ∗P < 0.05 vs. control). Figure S2: role of SIRT1 in the anti-inflammatory effects of CR in CIN. SD rats were pretreated with CR for 4 weeks. CIN was induced by intravenous injection of the CM iopromide (1.8 g/kg). The SIRT1 inhibitor EX527 (500 m/kg) was injected intravenously before establishment of CIN. The kidney samples were collected 24 h after CM injection. MPO activity (a) and IL-1β (b) and TNF-α (c) levels in the renal outer medulla were measured to determine the inflammatory response (∗P < 0.05 vs. control). The values are presented as mean ± standard deviation (n = 7, ∗P < 0.05 vs. control, #P < 0.05 vs. CM alone, &P < 0.05 vs. CM+CR). Figure S3: original western blot images. (a) and (b): refer to Figure 2(c). (c) and (d): refer to Figure 3(a). (e) and (f): refer to Figure 5(e). [file 2999296.f1.pdf]
